# Effectiveness of abdominal electroacupuncture therapy for poststroke constipation: a meta-analysis

**DOI:** 10.3389/fneur.2024.1480681

**Published:** 2024-11-22

**Authors:** Xiuzhen Zhao, Linxi Liu, Yingxiu Diao, Chunling Ma

**Affiliations:** ^1^The First Dongguan Affiliated Hospital, Guangdong Medical University, Dongguan, China; ^2^Guangzhou University of Traditional Chinese Medicine, Guangzhou, China; ^3^School of Rehabilitation Medicine, Gannan Medical University, Ganzhou, China

**Keywords:** stroke, constipation, electroacupuncture, rehabilitation, meta-analysis

## Abstract

**Background:**

Electroacupuncture (EA) has been employed to address the symptoms of constipation in individuals who have experienced a stroke. However, supporting evidence for its efficacy is lacking. This meta-analysis aims to investigate whether EA was effective in treating poststroke constipation.

**Methods:**

We conducted a comprehensive search of eight databases, including four English-language databases (PubMed, Embase, Web of Science, and the Cochrane Library) and four Chinese-language databases (Chinese Biomedical Literature Database, China National Knowledge Infrastructure, VIP, and Wanfang), for randomized controlled trials (RCTs) published from inception through January 31, 2023. To assess treatment effectiveness, we calculated the risk ratio (RR) or mean difference (MD) with 95% confidence interval (CI).

**Results:**

A total of 9 RCTs involving 601 participants were included. No heterogeneity was found across the included RCTs. The results of this meta-analysis indicated significant improvements in the total effective rate (RR = 1.39, 95% CI 1.27, 1.52), cure rates (RR 1.87, 95% CI 1.38, 2.54), constipation scoring system (MD = −2.51, 95% CI −3.05, −1.97), and quality of life (MD = −10.69, 95% CI −14.2, −7.17) in the EA group compared with the control group.

**Conclusion:**

Current evidence indicates that EA may be recommended for patients experiencing poststroke constipation. The lack of thorough investigations has undermined the quality of the major findings.

## 1 Introduction

Stroke is a neurological abnormality brought on by cerebrovascular conditions such as cerebral infarction, cerebral hemorrhage, and subarachnoid hemorrhage, which cause localized damage to the central nervous system (1). This illness places a considerable financial strain on society and healthcare systems, consistently ranking as one of the leading causes of mortality, disability, and public health issues globally (2). In comparison to patients in the acute stage, convalescent patients experience a higher incidence of constipation, which detrimentally affects their quality of life (QoL), physical functioning, and recovery capacity (3). Previous systematic reviews have shown that the prevalence of constipation in stroke patients is approximately 48% (95% CI: 33–63%) (4). Due to limitations in physical activity and other factors, the prevalence of movement disorders is higher in stroke patients compared to healthy individuals (5). Poststroke constipation not only impacts patients’ QoL but also makes it more difficult for patients to recover and can even lead to the recurrence of cerebrovascular diseases (6).

In recent years, research has primarily concentrated on the incidence of poststroke constipation, while effective management strategies for these patients remain scarce (4, 7). Gastrointestinal (GI) illnesses are highly prevalent worldwide. Due to recurrent symptoms and the sometimes limited effectiveness of conventional treatments, patients with gastroesophageal reflux disease (8), functional dyspepsia (9), irritable bowel syndrome (10), and inflammatory bowel conditions (11) often turn to complementary therapies, including acupuncture. For thousands of years, Asian nations have used acupuncture as a form of medicine. Over the past few decades, acupuncture has also drawn more attention in western nations, with numerous studies examining its function in gastroenterology (12). Current drug therapies are often inadequate in alleviating symptoms and enhancing the quality of life for patients with constipation. The physiopathology of constipation can be complex, with various underlying mechanisms potentially contributing to treatment resistance. Therefore, there is a pressing need for simple and accurate diagnostic tools to identify specific subtypes of constipation that may respond to targeted therapies (13, 14). Additionally, adverse effects such as bloating (15), diarrhea (16), and nausea (17) are frequently reported. Therefore, we need to urgently look for effective treatment strategies with fewer side effects.

Traditional Chinese medicine (TCM) has long been used in China and globally due to its safety and long-lasting effects (18, 19). As a key component of complementary medicine, acupuncture is gaining popularity in numerous medical institutions for treating patients with poststroke constipation (20). Electroacupuncture (EA) is a therapeutic technique that combines the use of needles and electrical stimulation to prevent and treat various diseases. Low-frequency pulsed current is applied near the body’s bioelectric pathways, with the goal of eliciting therapeutic effects through the modulation of the body’s electrical signals and energy flows (21). This particular remedy has been employed to address several ailments including diarrhea, and abdominal pain (22, 23). According to preclinical research, EA stimulation of abdominal points has been shown to impact sympathetic innervation of the gastrointestinal tract, whereas stimulation of limb points can influence parasympathetic input (23, 24).

A systematic review has recommended EA as a treatment for poststroke constipation (25), and one study has indicated that EA may have some long-lasting effects (26). However, whether this evidence is applicable to the treatment of poststroke constipation remains unclear. EA can also stimulate the parasympathetic nerve, increase rectal pressure, and restore the sense of defecation (16). Despite the longstanding use of acupuncture as a potential treatment modality, there have been no rigorous qualitative or quantitative analyses evaluating the effects of EA on the abdomens of patients with stroke for the management of constipation. Overall, there is a paucity of evidence supporting the efficacy and safety of EA in the treatment of poststroke constipation. In this systematic review, we sought to comprehensively evaluate the existing literature to assess the efficacy and safety of EA for the management of constipation in the stroke population.

## 2 Methods

### 2.1 Study registration

This systematic review and meta-analysis was conducted and reported in accordance with the Preferred Reporting Items for Systematic Reviews and Meta-Analysis (PRISMA) checklist (27) and the Cochrane Handbook for Systematic Reviews of Interventions (28). This study has also been registered on the PROSPERO website (ID: CRD42022375282).^1^

### 2.2 Database and search strategy

A comprehensive literature search was conducted across four English-language databases (PubMed, Embase, web of science, and the Cochrane central register of controlled trials) and four Chinese-language databases [Chinese biomedical literature database, China National Knowledge Infrastructure (CNKI), Chinese science and technology periodicals (VIP), and WANFANG digital periodicals (WANFANG)] for randomized controlled trials (RCTs) published from inception through January 31, 2023. The topic of this study is EA for the treatment of poststroke constipation; thus, the following medical subject words were used for the search: electroacupuncture AND stroke AND (constipation OR dysporia OR dysdefecation).

### 2.3 Study selection

Two independent reviewers (XZZ and LXL) conducted the study selection process, including screening of titles and abstracts, as well as assessing the full-text eligibility of potentially relevant studies. Any discrepancies were resolved through discussion or consultation with the corresponding author (CLM).

### 2.4 Type of studies

#### 2.4.1 Participants

Adults who met the diagnostic standards (29) for post-stroke constipation were included in this study, which used EA as an intervention technique. We included all participants, without any restrictions on site of injury, type of injury, degree of constipation, or timing of the stroke, to include all pertinent articles.

#### 2.4.2 Interventions

The main type of intervention included was the use of different forms of electrical stimulation to target the acupoints treating poststroke constipation. Relevant RCTs performing a comparison between EA plus conventional treatment (medication, manipulation, or any other treatment) and the same conventional treatment were included, regardless of blinding.

#### 2.4.3 Outcomes

The primary outcome measures included the total effective rate, cure rate, and QoL following electroacupuncture (EA) treatment. Additionally, the patients’ clinical scores and QoL were evaluated using standardized tools such as the Constipation Scoring System (CSS), Patient Assessment of Constipation Quality of Life (PAC-QoL), or other relevant scales.

### 2.5 Exclusion criteria

Studies were excluded based on the following criteria: (1) studies with inappropriate randomization methods or incomplete data; (2) reviews, case reports, non-clinical studies, and animal studies; (3) studies lacking relevant data of interest; (4) studies with unclear or insufficient outcome measures; and (5) multiple publications reporting identical findings.

### 2.6 Data extraction and management

To identify the eligible RCTs, two authors (YXD and XZZ) independently examined the full texts of all articles retrieved from the databases. A third researcher (CLM) helped settle any disagreements regarding the studies that were chosen. The reviewers selected the studies after reviewing the exclusion and inclusion criteria and rigorously analyzing the titles and abstracts of all studies; after reading the studies, they decided which reserved documents to include. For each eligible study, the following items were extracted independently: author, year, randomization method, sample size, patient age, disease course, intervention, treatment course, total effective rate, cure rate, and adverse event records. Any differences during the extraction process were resolved by principal investigator (CLM). If missing data were found, the corresponding author was contacted by email for information. In cases of several studies describing the same trial, only one study was included.

### 2.7 Risk of bias assessment

The study quality was independently assessed by two investigators (YXD and XZZ) using the Cochrane system-recommended ROB assessment tools (RevMan 5.4) (28). The assessment was based on the following criteria: random sequence generation and allocation concealment (selection bias), blinding of participants and outcome assessors, handling of incomplete results, selective reporting, and other potential sources of bias. Each criterion was rated as having a high, low, or unclear risk of bias (ROB). In case of any disagreement between the assessors, one of the corresponding authors (CLM) was consulted for resolution. The overall risk of bias was determined based on the ratings of each criterion.

### 2.8 Data synthesis and statistical analysis

The data analysis was performed using the RevMan 5.4 software developed by the Cochrane Collaboration in the United Kingdom. For the main results of the studies that were included, we computed aggregate estimates with 95% CIs using *RR* for count data and *MD* with 95% CIs for measurement data. A significance level of *p* < 0.05 was deemed to have statistical significance. The heterogeneity among the studies included in the analysis was evaluated using a heterogeneity test. If there was no notable variation (*I*^2^ < 50%), the fixed-effects model was employed to merge the effect estimates. If there was a substantial amount of variation (*I*^2^ > 50%), the random-effects model was used instead (49). Any potential outliers or influential studies were discovered and addressed through the use of sensitivity analysis. If the data contained in the study could not be subjected to meta-analysis, a descriptive analysis was performed. Publication bias was evaluated by generating funnel plots using RevMan 5.4. The PRISMA principles were adhered to during the execution of all these procedures (30).

## 3 Results

### 3.1 Study identification and selection

**Figure 1:**
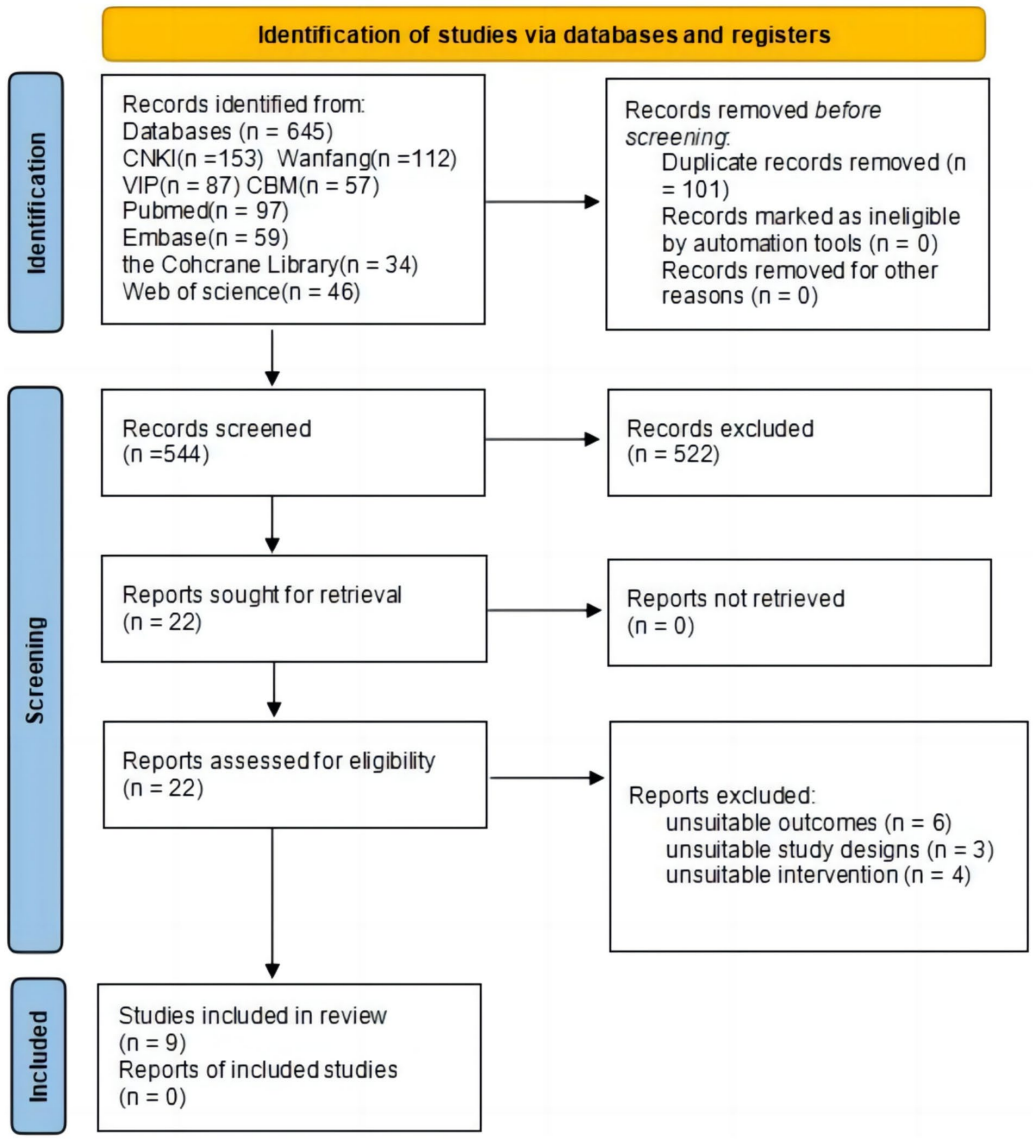
Flow chart of study search and selection process.

We conducted an extensive search across eight electronic databases and found a total of 645 studies using the preset search tactics. After eliminating duplicate entries, there were 544 studies remaining. Out of these, a total of 522 studies were eliminated after reviewing their titles and abstracts because they did not match the predetermined criteria for inclusion. After thoroughly examining the complete texts of the remaining 22 investigations, an additional 13 studies were eliminated due to the predetermined exclusion criteria. Therefore, a grand number of nine RCTs were incorporated into the meta-analysis (31–39). For a comprehensive depiction of the research selection and inclusion process, please consult (Figure 1).

### 3.2 Characteristics of the included studies

Table 1 presents the attributes of the participants and the frequency of undesirable incidents in the studies that were included. The study included nine RCTs (31–39) with a total of 601 patients (344 men and 257 women). Among them, 315 patients were in the EA group and 286 patients were in the control group. The nine investigations were carried out only in China from 2008 to 2017, and the duration of the follow-up periods varied from 13 to 90 days. The experimental groups were administered conventional EA, while the control groups received either filiform needle acupuncture or medicine. All of the trials documented both the overall efficacy rate and the rates of complete recovery. Eight RCTs (31–37, 39) utilized the CCS to evaluate the patients’ scores, while only two investigations employed the PAC-QoL scale to examine the QoL. Table 2 provides a summary of the interventions’ characteristics, which include the treatment equipment, point selection site, and treatment prescription. The acupoints Tianshu (ST 25) and Daheng (SP 15) were the most commonly utilized in the investigations, with Tianshu being used in 88.9% of the studies (31–34, 36–39) and Daheng in 66.7% of the studies (31–35, 38, 39). The duration of the treatment varied between 2 and 4 weeks, with sessions occurring 6–7 times each week, lasting 30 min each. Table 3 provides a concise overview of the research’ objectives, criteria for inclusion, and key findings.

**Table 1:** Basic characteristics of EA treatment of poststroke constipation.

Study	Study design	Duration (day)	Age, years (mean ± SD)	Interventions	Sample size	Gender		Follow-up (day)	Outcome measures	Adverse effects
						Female	Male			
Wang et al. (31)	RCT	5.75 ± 1.25 5.65 ± 1.09	63.2 ± 3.74 61.9 ± 4.6	EA/Medication	80	35	45	16	Total effective rate Cure rate CCS	None
Zhu (39)	RCT	61.77 ± 18.85 67.17 ± 20.68	57.2 ± 8.01 56.8 ± 8.16	EA/Filiform needle	60	23	37	15	Total effective rate Cure rate CCS	None
Jiang (33)	RCT	60.6 ± 14.5 62.8 ± 13.57	54.4 ± 6.98 53.6 ± 6.95	EA/ Filiform needle	60	24	36	13	Total effective rate Cure rate CCS	None
Jin (34)	RCT	3.87 ± 1.995 4.2 ± 1.972	53.57 ± 5.191 52.4 ± 4.789	EA/Medication	60	30	30	28	Total effective rate Cure rate CCS PAC-QoL	None
Yin et al. (38)	RCT	N/A	44.25 ± 9.24	EA/Medication	78	36	42	14	Total effective rate Cure rate	None
Peng and Li (37)	RCT	49.58 ± 5.61 47.82 ± 5.37	51 ± 6 53 ± 7	EA/Medication	48	26	22	14	Total effective rate Cure rate CCS	None
Li (35)	RCT	64.47 ± 14.53 62.67 ± 17.43	65.13 ± 9.18 63 ± 8.23	EA/ Filiform needle	60	35	25	14	Total effective rate Cure rate CCS	None
Liu et al. (32)	RCT	3–7 3–8	54 56	EA/Medication	68	31	37	14	Total effective rate Cure rate CCS	None
Lu (36)	RCT	N/A	63.75 ± 10.885 64.69 ± 11.031 61.83 ± 9.337	EA/Medication	87	17	70	90	Total effective rate Cure rate CCS PAC-QoL	None

**Table 2:** Characteristics of EA parameters.

Study	Instrument	Target (international code)	Acupuncture		Frequency/Intensity	Treatment time (min/session, sessions/week, week)
			Diameter (mm)	Length (mm)		
Wang et al. (31)	G6805-2A EA instrument (Produced by Shanghai Huayi Medical Instrument Factory)	Daheng (SP15) Fujie (SP14) Tianshu (ST25) Shuidao (ST28)	0.4	50	80 ~ 100 times/min	30 min/s, 7 s/w, 2w
Zhu (39)	N/A	Daheng (SP15) Fujie (SP14) Tianshu (ST25) Shuidao (ST28) Guilai (ST29) Piyu (BL20) Qihai (RN6)	0.3	40	Abdominal muscles tighten and move up and down with the handle	30 min/s, 6 s/w, 2w
Jiang (33)	KWD-808II pulse electrotherapy instrument	Daheng (SP15) Fujie (SP14) Tianshu (ST25) Shuidao (ST28) Fuai (SP16) Jianli (RN11)	0.3	40	Abdominal muscles tighten and move up and down with the handle	30 min/s, 6 s/w, 2w
Jin (34)	G6805 EA instrument	Daheng (SP15) Tianshu (ST25) Shuidao (ST28) Guilai (ST29) Guanyuan (RN4) Zhongwan (RN12) Zhigou (SJ6)	N/A	N/A	The intensity was determined by the patient’s abdominal muscle contraction and tolerance	30 min/s, 6 s/w, 4w
Yin et al. (38)	Han’s EA Instrument (LH202H)	Daheng (SP15) Fujie (SP14) Tianshu (ST25) Shuidao (ST28)	0.4	50	2 Hz/15 Hz	30 min/s, 7 s/w, 2w
Peng and Li (37)	Type G6805-II EA instrument	Tianshu (ST25)	0.3/0.3	25/75	N/A	30 min/s, 6 s/w, 2w
Li (35)	KWD808I pulse therapeutic instrument	Daheng (SP15) Guanyuan (RN4) Zhongwan (RN12)	0.3	40	The patient’s abdominal muscles are tightened with slight tremor	30 min/s, 7 s/w, 2w
Liu et al. (32)	KWD808-I EA therapeutic instrument	Tianshu (ST25) Zhongwan (RN12) Zusanli (ST36) Shangjuxu (ST37) Xiajuxu (ST39)	0.3	40	2 Hz	30 min/s, 7 s/w, 2w
Lu (36)	Nanjing Komatsu XS 998804 EA instrument	Zhigou (SJ6) Tianshu (ST25) Shangjuxu (ST37)	N/A	N/A	The intensity is tolerated by the patient	30 min/s, 6 s/w, 2w

**Table 3:** The aim, primary findings, and conclusions of the studies included in this systematic review.

Study	Aim	Inclusion criteria	Exclusion criteria	Main results	Conclusions
Wang et al. (31)	To assess the effectiveness of abdominal EA compared to western therapy in treating constipation following a stroke.	(1) Patients who fulfill the diagnostic criteria for stroke and exhibit positive CT or MRI findings; (2) Patients who fulfill the diagnostic criteria for constipation; (3) Patients who have stable vital signs following a stroke; (4) Individuals aged between 35 and 70 years; (5) Patients who are admitted to the hospital	(1) Organic gastrointestinal lesions, such as inflammatory bowel disease, intestinal tuberculosis, colon polyps, etc.; (2) Patients with a history of gastrointestinal surgery; (3) Patients with severe underlying diseases and psychiatric disorders; (4) Patients with severe cardiac, hepatic, and renal impairment; (5) Patients who are unwilling to undergo this treatment.	The overall efficacy rate of 92.5% in the EA group was substantially superior to the efficacy rate of 72.5% in the medication group (*P* < 0.05).	Abdominal EA has a clear clinical impact on enhancing the movement of the gastrointestinal tract and alleviating constipation in stroke patients suffering from constipation.
Zhu (39)	The objective of this study is to examine the therapeutic impact of abdominal EA on postapoplexy deficiency and the associated changes in clinical symptoms before and after treatment. The aim is to offer an effective and easy therapy option for managing postapoplexy deficiency and its related syndrome.	(1) Patients who integrate the diagnostic criteria of both Western medicine and Chinese medicine in relation to the upper brain; (2) The merging of upper convenience esoteric diagnosis based on medical standards and deficiency esoteric syndrome types; (3) Age-related mortality after the stabilization of bodily indicators; (4) Normal defecation in the frontal region of the brain, with no previous record of secretive defecation; (5) The state of being fully conscious and alert, sufficient for appropriate treatment.	(1) Patients with transient ischemic attack (TIA); (2) It has been confirmed that the stool is caused by tumors, direct bowel issues, and lesions at the junction; (3) Retention of gastric tube and liquid food; (4) Identify and acknowledge the barriers of knowledge that prevent the normal flow of progress; (5) In the week prior to treatment, patients experience intestinal obstruction, difficulty in defecation, and require medication; (6) The disease progression is worsening; (7) Combined with severe cardiovascular, biliary, hepatic, renal, and hematopoietic system disorders.	The difference in symptom scores between the two groups before and after treatment was statistically significant (*P*<0.05). Additionally, both the control group and the treatment group experienced improvements in constipation symptoms.	Both the treatment group and the control group exhibited a distinct impact on the management of postapoplexy deficit. The therapy group had a significant impact on the occurrence of apoplexy.
Jiang (33)	The objective of this study was to examine the clinical impact of abdominal EA in treating post-stroke qi secretions and to assess the changes in clinical symptoms of qi secretions before and after therapy.	(1) Fulfill the diagnostic criteria for both ischemic and hemorrhagic stroke. (2) Fulfill the diagnostic criteria for constipation. (3) Patients suffering from constipation who fulfill the diagnostic criteria for TCM constipation and TCM syndrome type criteria. (4) Individuals aged 40 to 65. Patients who have experienced a stroke and have stable vital signs.	(1) Stroke does not cause constipation. (2) Patients aged between 40 and 65 years. (3) The patient is experiencing severe cardiac insufficiency (grade IV or above), respiratory circulatory failure, and severe liver and renal insufficiency. (4) Prolonged use of cisapride, ketamine, and other forms of symptomatic therapy can lead to drug dependence. (5) Patients who are unable to tolerate EA treatment. (6) All individuals who had already experienced constipation prior to their stroke.	The symptom score ratio between the two groups showed a significant difference (*P*<0.05), suggesting that the effectiveness of the two groups was better after two courses of treatment compared to after one course of treatment. Additionally, the effectiveness of abdominal EA was more pronounced.	(1) Both abdominal EA and conventional acupuncture have been found to be beneficial in the treatment of constipation following a stroke. (2) Abdominal EA has shown superior efficacy compared to the conventional acupuncture group in the treatment of poststroke constipation.
Jin (34)	The pursuit of an effective treatment plan for postapoplexy constipation was facilitated by the comparison of the effective effect of EA stimulation of abdominal acupoints in combination with medications.	(1) Males and females between the ages of 40 and 70; (2) Stroke (ischemic and hemorrhagic) that meets the diagnostic criteria of both Chinese and Western medicine; (3) Constipation that meets the diagnostic criteria. (4) The patient’s vital signs were stable. (5) The patient was capable of autonomously completing the scale assessment, possessed of communication and expression skills, and was clear and articulate. (6) The subject signed informed consent, either personally or through his immediate family members.	(1) Stroke does not cause constipation. (2) Patients who are younger than 40 years old or older than 70 years old. (3) Patients who have serious primary diseases such as cardiovascular, liver, kidney, digestive, hematopoietic, or other conditions. (3) Patients with mental illnesses who are prone to infection, bleeding, or have allergic tendencies. (4) Patients who have developed drug dependence due to long-term use of cisapride, ketamine, or other symptomatic treatment drugs.	The abdominal acupuncture coupled with medicines group and the oral medication group showed statistically significant changes in clinical scores, QoL assessment, and TCM symptom ratings of patients with constipation before and after therapy (*P*<0.05).	(1) The combination of abdominal EA with drug therapy and oral medication alone may effectively alleviate constipation symptoms in patients after a stroke. (2) Abdominal EA coupled with drug therapy is more effective than oral medication alone in treating constipation after a stroke.
Yin et al. (38)	The objective of this study is to assess the effectiveness of EA in combination with bifidobacterium for treating constipation after acute cerebral apoplexy.	(1) A minimum of 25% of bowel movements are difficult; (2) A minimum of 25% of defecation consists of dry ball stool or hard stool; (3) A minimum of 25% of defecation feels incomplete; (3) A minimum of 25% of defecation feels like there is obstruction in the anal rectum; (5) A minimum of 25% of defecation requires manual assistance, such as using fingers or pelvic floor support. (6) Bowel movements occur three times every week.	(1) Organic gastrointestinal lesions; (2) Severe cardiovascular, hepatic, renal, cerebral, and hematological disorders; (3) Previous gastrointestinal surgical procedures.	The overall efficacy rate of the combination group was 87.18%, which was considerably superior to that of the medication group (64.10%) (*P* < 0.05).	The combination of EA and pharmacological therapy is an effective and clinically applicable treatment for poststroke constipation.
Peng and Li (37)	The objective of this study is to examine the therapeutic impact of deep acupuncture at the Tianshu point on poststroke constipation.	(1) Fulfill the stroke diagnostic criteria outlined in the Guidelines for Clinical Research of New Chinese Medicines (2002 edition); (2) Satisfy the diagnostic criteria for functional constipation Roman III, as established by the American Gastroenterological Board and published in May 2006; (3) Individuals aged 36-65 years; (4) Patients who have been admitted to the hospital; (5) Patients who have had a stroke and have stable vital signs; (6) Patients or their family members who have given informed permission.	(1) Individuals with colon polyps, inflammatory bowel disease, intestinal tuberculosis, and other significant organic intestinal diseases; (2) Individuals with a history of digestive tract surgery; (3) Individuals with severe cardiac, liver, and renal impairment; (4) Constipation caused by other factors; (5) Individuals with severe underlying diseases and mental disorders; (6) Those who discontinue treatment prematurely.	Following the completion of one treatment course, the clinical effectiveness was assessed. The symptom score, overall effective rate, significance rate, and above significance rate of patients in the EA group were superior than those in the medication group, and the difference was statistically significant (*P* < 0.05).	EA administered at the Tianshu point has been shown to greatly enhance the clinical outcomes and overall QoL for individuals suffering with post-stroke constipation.
Li (35)	The objective of this study is to evaluate the therapeutic efficacy of abdominal EA in managing constipation after a stroke, and to provide a more streamlined, user-friendly, and efficient treatment approach for post-stroke constipation.	(1) Patients who satisfy the diagnostic criteria for poststroke constipation; (2) Individuals between the ages of 40 and 75; (3) In patients recovering from stroke with constipation, the duration of stroke was 2-6 months; (4) The vital signs were stable following the stroke (including disorders such as unconsciousness, aphasia, and hearing impairment); (5) Individuals who are willing to undergo the treatment protocol outlined in this research.	(1) Individuals experiencing TIA; (2) Constipation resulting from organic conditions such as intestinal stenosis and blockage caused by rectal and colon disorders (e.g., tumors, Crohn’s disease, colon polyps, intestinal TB, etc.). (3) Patients with severe cardiovascular, liver, renal, and hematological system disorders, cancer, dementia, and mental illness; (4) Patients with a gastric tube and liquid diet due to medical conditions; (5) Individuals who are unable to collaborate with acupuncture therapy or have an unstable condition	Following a 14-day treatment period, the treatment group exhibited a total effective rate of 96.67%, whereas the control group had a rate of 86.67%. The observed difference between the two groups was statistically significant (*P*<0.05).	Both abdominal EA and traditional acupuncture have been shown to be successful in treating poststroke constipation. However, abdominal EA has been shown to be superior than conventional acupuncture in terms of its effectiveness.
Liu et al. (32)	The objective of this study is to examine the therapeutic impact of the combined use of acupuncture and moxibustion on post-apoplexy constipation.	The diagnostic criteria for stroke are based on the guidelines outlined in the Diagnostic Essentials of Various Cerebrovascular Diseases, which were established at the Fourth National Conference on Cerebrovascular Diseases. Stroke diagnosis is verified by the use of head CT and/or MRI scans. The diagnostic criteria for constipation are based on the criteria outlined in the Guiding Principles for Clinical Research of New Chinese Medicines, which were established by the Ministry of Health in 1993.	(1) Organic gastrointestinal abnormalities, such as inflammatory bowel disease, intestinal tuberculosis, colon polyps, etc.; (2) Patients with a history of gastrointestinal surgery; (3) Patients with severe underlying diseases and mental disorders; (4) Patients with severe cardiac, liver, and renal impairment; (5) Patients who are unwilling to undergo this treatment.	Following therapy, the clinical scores of constipation in both groups decreased compared to the ratings before to treatment. These changes were found to be statistically significant (*P* < 0.01). The treatment group had a total effective rate of 80.0%, whereas the control group had a rate of 72.7%. The disparity between the two groups was statistically significant (*P* < 0.05).	Acupuncture and moxibustion are efficacious techniques for managing post-apoplectic constipation.
Lu (36)	By observing the clinical efficacy of deep acupuncture at Tianshu point in branch groove of EA and western medicine in the treatment of poststroke constipation, the QoL of poststroke constipation patients can be greatly improved, and a better treatment method for poststroke constipation can be provided.	(1) Aged between 35 and 80 (including 35 and 80), both male and female. (2) It was consistent with the diagnostic standards of Chinese and western medicine for stroke. (3) In line with the standards of Chinese and Western medicine for constipation. (4) Stable vital signs and clear consciousness. (5) Normal hearing and understanding, can cooperate with treatment. (6) Accept the study and sign the informed consent.	(1) Organic lesions of colon and rectum (such as tumors and colon polyps). (2) Patients with serious complications such as heart failure, kidney failure, etc., or with malignant tumors. (3) Patients with gastric tube. (4) Cases that did not meet the inclusion criteria.	The curative effect of Huishu group was better than Shangjuxu Jigou group and Western medicine group, and there was statistical significance (*p* > 0.05).	EA deep acupuncture Tianshu point is significant in the treatment of poststroke constipation. Compared with Shangjuxu, Zhigou point and Western medicine, EA can improve clinical symptoms more effectively, and has the advantages of safety and low recurrence rate.

EA, Electroacupuncture; TCM, Traditional Chinese Medicine; CT, Computed Tomography; MRI, Magnetic Resonance Imaging; QoL, Quality of Life; TIA, Transient Ischemic Attack.

### 3.3 Methodological quality of the included trials

**Figure 2:**
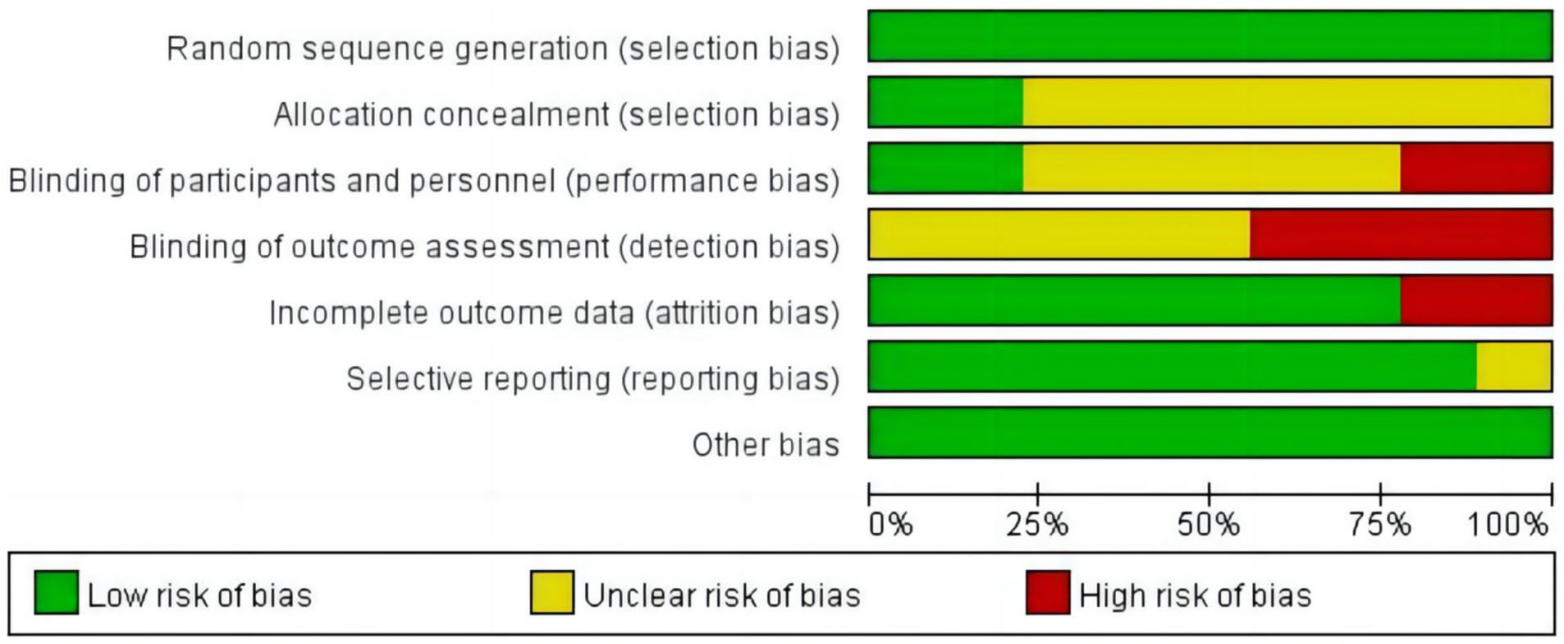
Risk of bias summary.

A risk assessment was performed to evaluate the methodological rigor of the studies that were included. Figure 2 presents the specific information regarding the assessment. All of the included experiments met the criterion for randomized sequence generation. Five studies (31, 32, 36–38) had an uncertain level of danger for both participants and assessors, while four studies (33–35, 39) had a high level of risk, and the quality of evaluations in these areas was subpar. Regarding data integrity, seven studies (31–36, 39) were evaluated as having a low risk, while the remaining two (37, 38) were considered to have a high risk. None of the studies revealed any additional bias.

### 3.4 Meta-analysis outcomes

#### 3.4.1 Total effective rate

**Figure 3:**
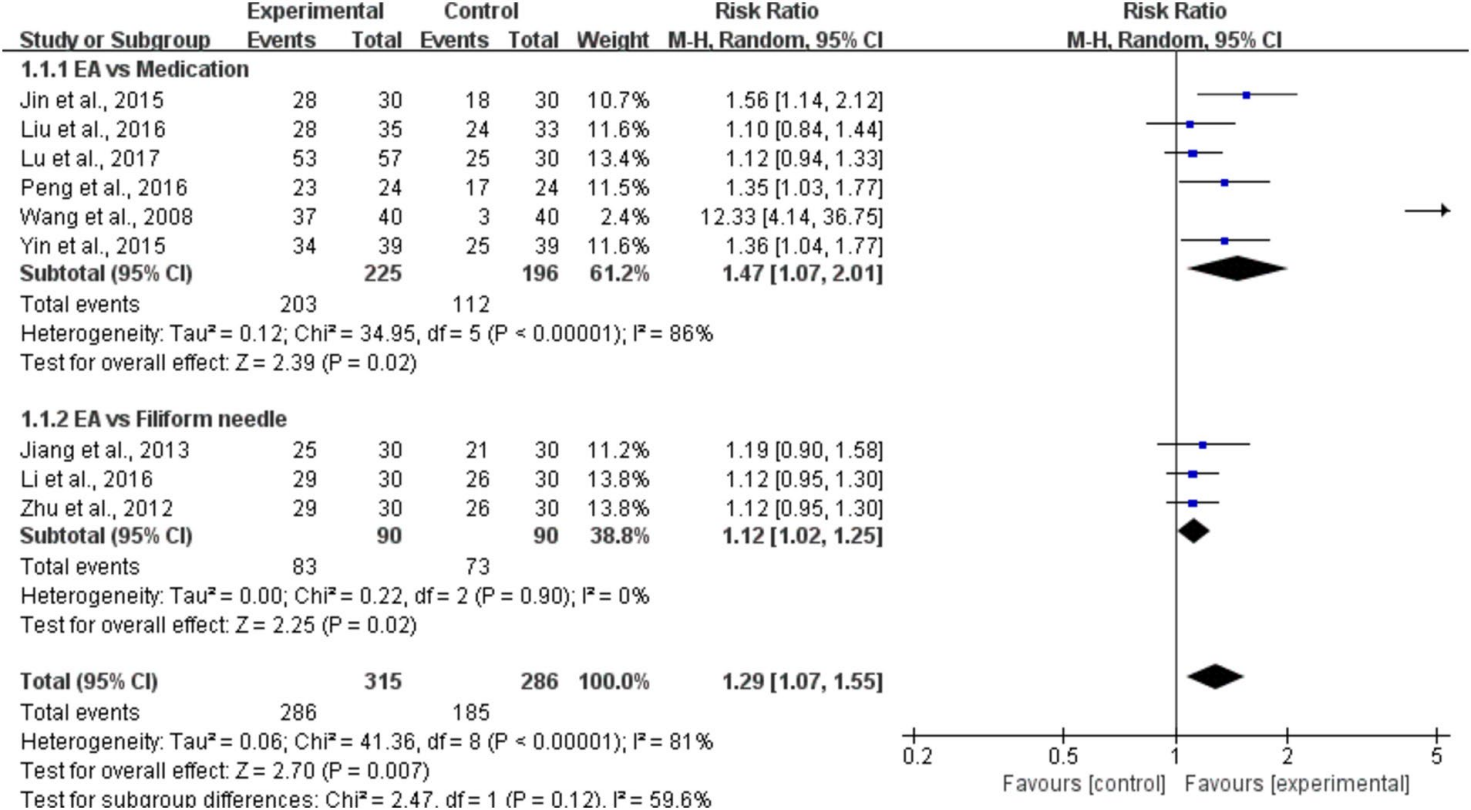
A forest plot of the subgroup analysis of the included studies comparing EA group and control group in changes of the total effective rate.

All nine studies provided information on the overall efficacy of EA in comparison to conventional treatment. Subgroup analysis was performed based on the outcome measures used as the criteria for grouping. The findings of the heterogeneity test suggested that the EA may be the main cause of heterogeneity. The findings demonstrated that EA is a highly successful treatment for poststroke constipation, as illustrated in Figure 3. The study included nine RCTs (31–39), with a total of 601 patients. These trials evaluated the overall effectiveness of EA in patients with poststroke constipation, comparing it to healthy individuals. The combined findings of these studies indicated that EA had a significant positive effect on the overall effective rate (RR = 1.29, 95% CI = [1.07, 1.55], *I*^2^ = 81%, *p* < 0.05). The subgroup analyses of the EA were conducted to compare the effectiveness of EA versus medication. The results showed a RR of 1.47, with a 95% CI ranging from 1.07 to 2.01. The heterogeneity of the studies, as measured by *I*^2^, was 86%. The *p*-value was less than 0.001, indicating statistical significance. The EA vs. filiform needle comparison yielded a RR of 1.12, with a 95% CI of [1.02, 1.25]. The heterogeneity (*I*^2^) was 0% and the p-value was less than 0.05. This information is presented in Figure 3.

#### 3.4.2 Cure rate

**Figure 4:**
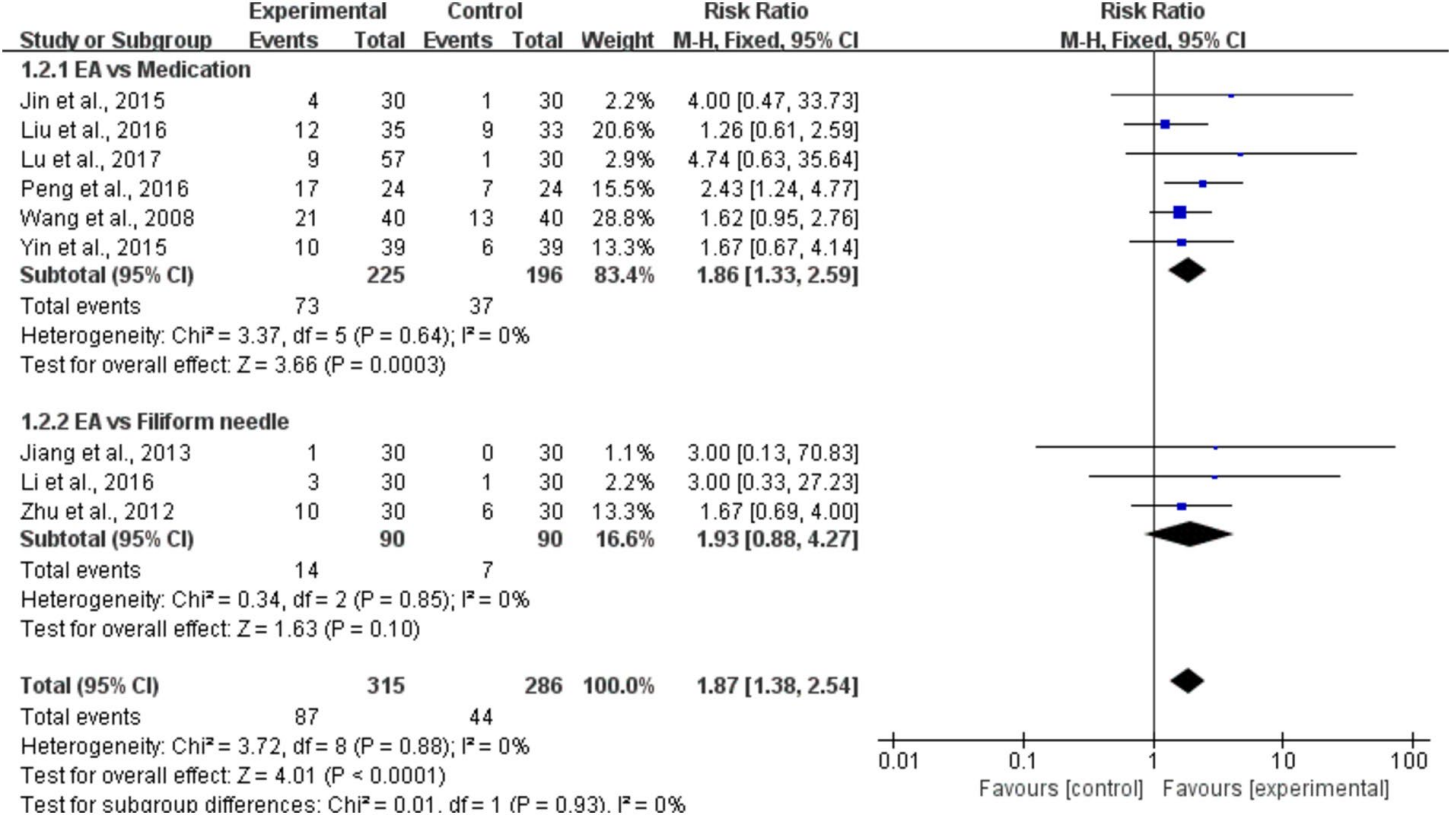
A forest plot of the subgroup analysis of the included studies comparing EA group and control group in changes of the cure rate.

We conducted subgroup analyses to further investigate the effect of EA on cure rates in patients with poststroke constipation compared to healthy individuals (31–39). The aggregated results demonstrated that EA significantly improved cure rates overall (*RR* = 1.87, 95% CI = [1.38, 2.54], *I*^2^ = 0%, *p* < 0.001), indicating a nearly twofold increase in the likelihood of cure with EA when compared to control treatments. In the subgroup analysis comparing EA to medication, the results also showed a significant improvement in cure rates for EA (*RR* = 1.86, 95% CI = [1.33, 2.59], *I*^2^ = 0%, *p* < 0.001). This suggests that EA is more effective than medication in improving cure rates, with an 86% higher likelihood of cure in the EA group. However, for the subgroup comparing EA to the use of filiform needles (a type of acupuncture technique), the results were not statistically significant (*RR* = 1.93, 95% CI = [0.88, 4.27], *I*^2^ = 0%, *p* > 0.05). Although the relative risk suggests that EA might be more effective than filiform needle therapy, the wide confidence interval and the *p*-value greater than 0.05 indicate that this result is not conclusive, and more studies would be necessary to determine if EA truly surpasses filiform needle therapy in terms of cure rates (Figure 4).

#### 3.4.3 CCS

**Figure 5:**
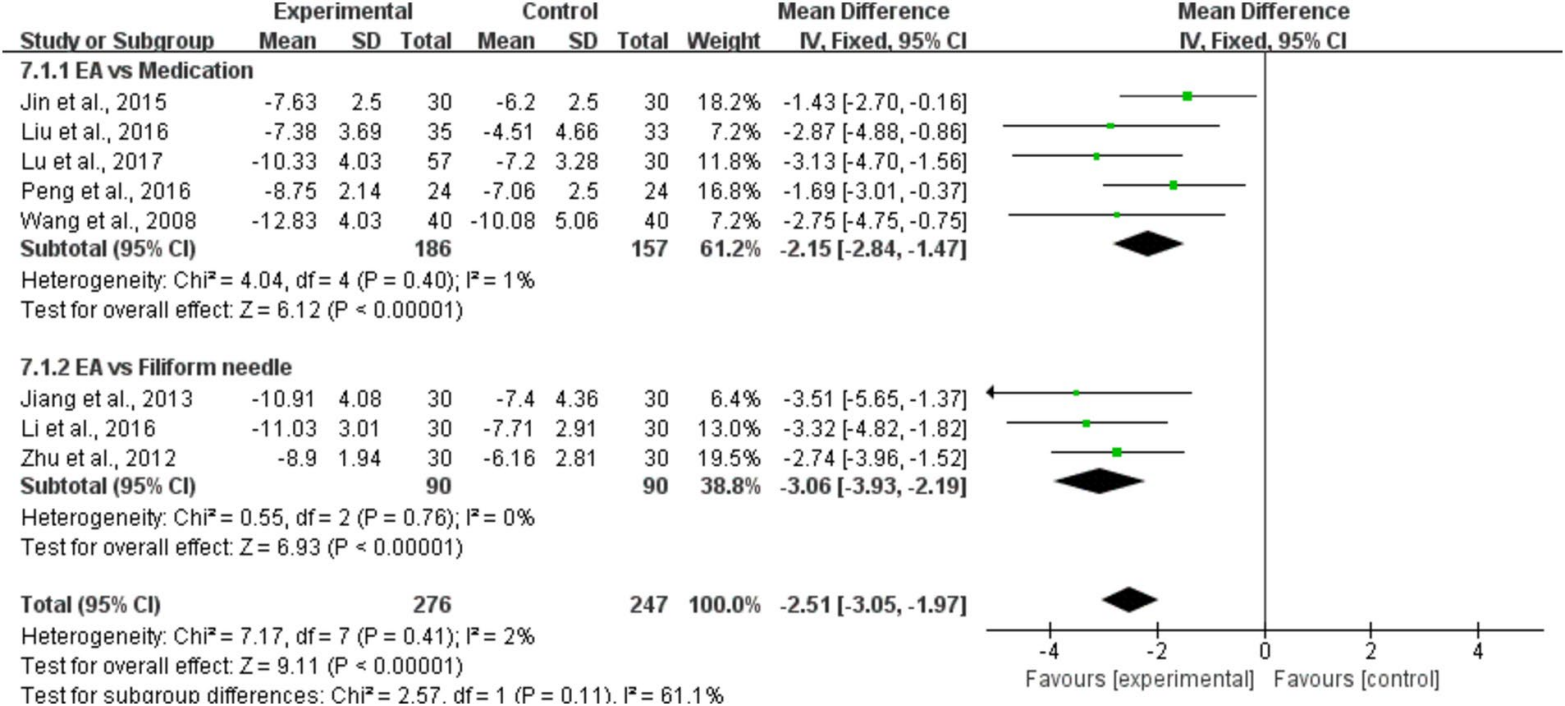
A forest plot of the subgroup analysis of the included studies comparing EA group and control group in changes of the CCS.

Eight studies (31–39), which included a total of 523 subjects, used the CCS to evaluate patients’ clinical outcomes. The aggregated results of these studies suggested that EA significantly improved the constipation score (*MD* = −2.51, 95% CI = [−3.05, −1.97], *I*^2^ = 2%, *p* < 0.001). Subgroup analyses of the EA, for the subgroup of EA vs. medication (*MD* = −2.15, 95% CI = [−2.84, −1.47], *I*^2^ = 1%, *p* < 0.001). For the EA vs. filiform needle (*MD* = −3.06, 95% CI = [−3.93, −2.19], *I*^2^ = 0%, *p* < 0.001) (Figure 5).

#### 3.4.4 PAC-QoL

**Figure 6:**
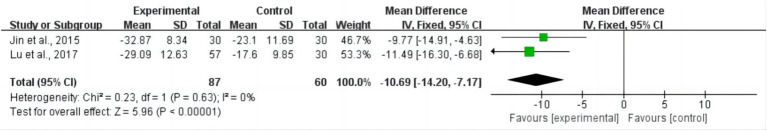
A Forest plot of EA group vs. control group: PAC-QoL.

The QoL in the target population was reported in just two research (34, 36). A grand number of 147 patients were evaluated. The QoL analysis utilized a fixed-effects model due to the absence of heterogeneity. The findings demonstrated that EA had a substantial positive impact on the QoL of patients with poststroke constipation, as compared to the control group (*MD* = −10.69, 95% CI = [−14.2, −7.17], *p* < 0.001) (Figure 6).

#### 3.4.5 Adverse events

None of the studies showed any negative effects in the treatment of poststroke constipation, suggesting that EA can be utilized as a secure and efficient traditional Chinese medicine approach in the therapeutic management of poststroke constipation.

#### 3.4.6 Publication Bias

**Figure 7:**
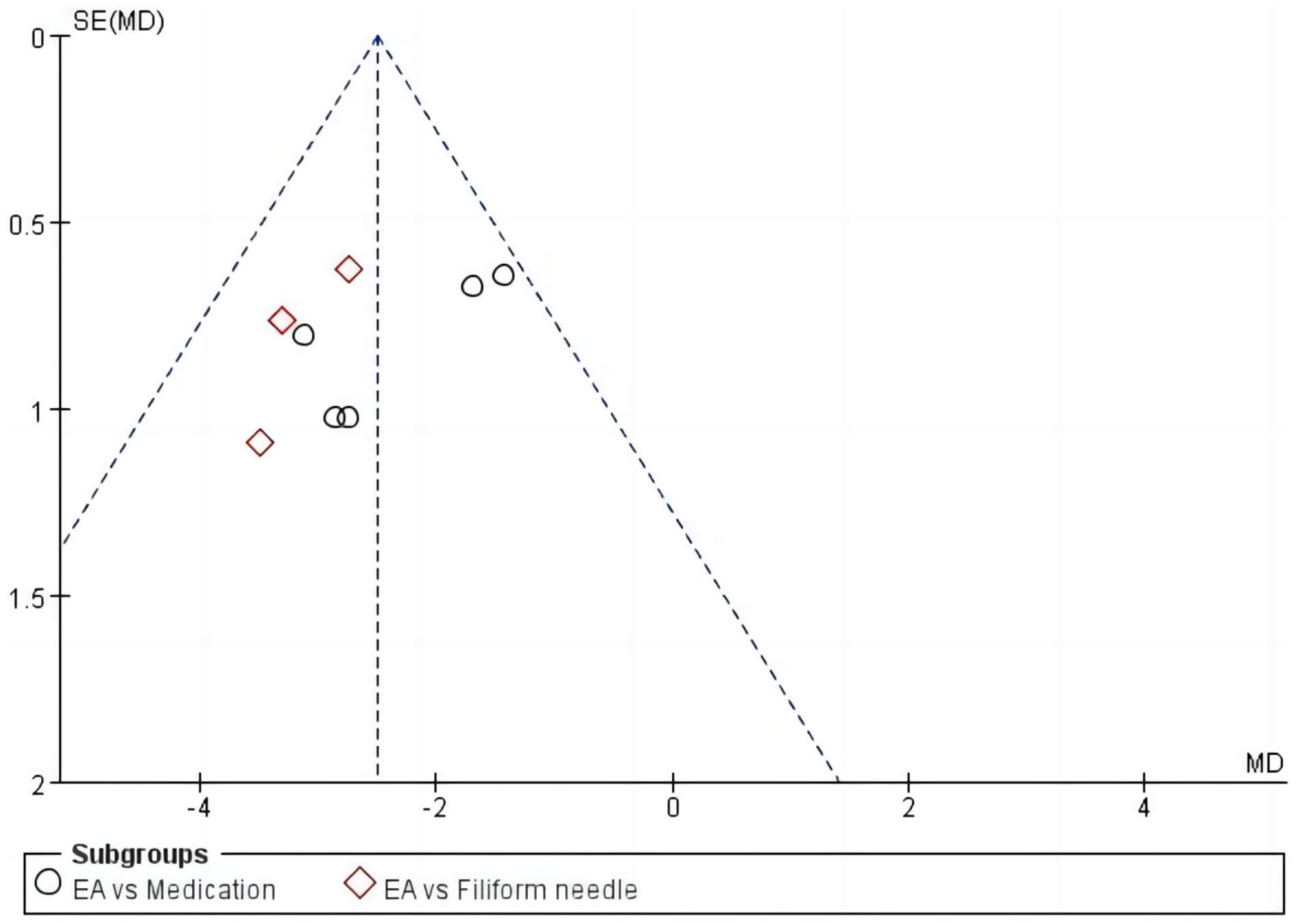
Funnel chart of clinical efficacy.

The RevMan 5.4 software was utilized to generate funnel plots for the identification of publication bias (Figure 7). The clinical efficacy analysis results and funnel plots indicated the presence of asymmetry and significant bias.

## 4 Discussion

This study is the initial comprehensive research and meta-analysis to consolidate the findings on the therapeutic impact of EA in individuals suffering from poststroke constipation. We conducted a thorough assessment of 9 randomized controlled trials (RCTs) in order to assess the effectiveness and safety of EA treatments in patients suffering from constipation after a stroke. Our study revealed a shortage of high-quality RCTs investigating therapies in this area. Tianshu (ST 25) and Daheng (SP 15) have been commonly chosen as acupoints for treating constipation in numerous investigations, resulting in positive therapeutic outcomes. As a result, they are favored in clinical practice.

When analyzing the results of the meta-analysis on the efficacy of abdominal EA therapy for poststroke constipation, it is crucial to take into account potential sources of bias and limitations in the studies that were included. Publication bias, selective reporting bias, and financing prejudice are potential causes of bias that could have impacted the overall findings. These biases can result in an inflated evaluation of the treatment impact and manipulate the body of data. Furthermore, it is important to consider constraints such as limited sample sizes, variations among the research, and the possibility of publication bias. These factors can influence the accuracy and applicability of the findings.

According to current medical theory, the objective of acupuncture treatment for poststroke constipation is to reestablish the patient’s physiological equilibrium by controlling the enteric nervous system, sympathetic and parasympathetic nervous systems, and the central nervous system (40). Multiple investigations have demonstrated that acupuncture has the ability to modulate the gastrointestinal functions of the body (41, 42). The afferent impulse of acupuncture travels to the brain centers at all levels, through the spinal cord, and out through the autonomic nervous system and humoral pathways to affect gastrointestinal function via the somatic nerve and vascular wall nerve plexus pathways (6, 43).

In order to improve the body’s ability to self-regulate and potentially expedite the healing process, certain sites along the meridians are physically stimulated by methods such as needle insertion, heat application, or pressure. Most acupuncture methods utilize single-use stainless-steel needles, each of which has a little larger diameter than the average human hair. The needles penetrate the “acupuncture points” on the skin. The needle that is inserted can be subjected to heat or electrical current, rotated, or moved vertically and horizontally at different rates and depths. The precise method by which EA affects poststroke constipation is not yet understood. Several factors, including as the mix of acupoints, meticulous selection of acupoints, and the depth of EA, can influence the effectiveness of EA. Further research and network meta-analyses are necessary to investigate the efficacy of different EA approaches. There is no medical treatment that can entirely erase the subjective sensations of patients. Conducting placid acupuncture is necessary to validate the impartiality of EA’s efficacy. Prior research has indicated that abdominal EA activates particular acupuncture points, such as Quchi (LI 11) or Shangjuxu (ST 37), which can regulate the equilibrium between the parasympathetic and sympathetic nervous systems. It is theorized that this stimulation enhances the movement of the gastrointestinal tract, therefore relieving symptoms of constipation. Additional research on the neurophysiological and biochemical mechanisms associated with abdominal EA therapy could enhance our understanding of how it works.

According to the findings of this meta-analysis, we saw significant improvements in the total effective rate, cure rate, CCS score, and PAC-QoL. These results provide confirmation of the usefulness of EA to a certain degree. This procedure may entail several treatment strategies. EA can enhance the patients’ symptoms through many means. Cui and colleagues conducted a study using 50 male Wistar rats to investigate the impact of various EA frequencies (ST25) on the time it takes for the first black stool to be excreted, as well as the electromyography of the colon, vasoactive intestinal peptide (VIP), and substance P immune activity in rats with slow transit constipation (44). The down-regulation of VIP immune activity and up-regulation of substance P immune activity in the colon tissue may have caused the electromyographic observation that EA enhanced colon activity. The therapeutic effects of 100 Hz-EA were less effective compared to those of 2 Hz/100 Hz-EA and 2 Hz-EA. In a separate research investigation, Liang and his team divided a total of 50 mice into two distinct groups. The first group, known as the model group (*n* = 40), did not get any form of treatment. On the other hand, the second group, referred to as the EA group, was subjected to a frequency range of 2–15 Hz with an amplitude of 1 mA at ST 37 for a duration of 15 min every day over a span of 3 consecutive days (45). An additional six mice formed a control group that did not experience constipation. This work provides evidence that the application of EA stimulation at acupoint ST 37 can partially reinstate the functionality of enteric neurons and ameliorate the impairment of intestinal motility. The enteric nervous system can impact changes in intestinal motility by affecting inhibitory neurons. In another study, Zhu and colleagues employed immunohistochemistry methods to identify the presence of protein gene product 9.5 and neuronal nitric oxide synthase in the muscle tissue of the jejunum, ileum, and proximal colon. Electrically activating acupuncture point ST 37 has been found to alleviate intestine motor dysfunction and partially restore intestinal neuron function, according to the research findings (43). The enteric nervous system can influence alterations in intestinal motility by impacting inhibitory neurons. The preceding description supports our findings, which suggest that using EA to stimulate the Tianshu, Daheng, and Shangjuxu points may be a more effective treatment for poststroke constipation in patients. Nevertheless, variations in the research findings may arise due to the limited duration of the follow-up periods and the absence of conscious analysis.

Due to the poor quality of the studies included and the overall lack of high-quality research on the use of EA for treating poststroke constipation, the findings of our analysis cannot be substantiated by the available literature. In addition, none of the studies included in the analysis have been registered in the Chinese Clinical Trial Registry, which prevents us from determining whether there has been any selective reporting of the study results. The randomization procedures, double blinding, adverse responses, and follow-up of the included studies exhibit significant faults, rendering the assessment of long-term treatment outcomes unfeasible. Furthermore, it is highly probable that there is selection bias, implementation bias, and measurement bias in the process due to the lack of universal diagnostic and efficacy evaluation criteria utilized in the literature, and the failure to adhere to internationally accepted standards (46). Effective use of blinding or masking techniques can significantly mitigate measurement bias and placebo effects. Out of the research that were analyzed, the double-blind principle was determined to have an unclear level of risk in 5 studies (31, 32, 36–38), high risk in 2 studies (33, 35) and low risk in 2 studies (34, 39). In five of the studies (31, 32, 36–38), the assessors were blind assessed as unknown risk. Due of the crucial role of communication between the therapist and patient in acupuncture, achieving double blindness is challenging.

A thorough and systematic review and analysis (47), pertaining to our research, was carried out to assess the efficacy and safety of acupuncture in treating constipation following a stroke. The study incorporated several RCTs and using the RoB 2.0 tool to evaluate the methodological quality. The meta-analysis was conducted using RevMan 5.3 and Stata 15.1 software, and the quality of evidence was evaluated using the GRADE approach. In addition, the study employed the Standards for Reporting Interventions in Clinical Trials of Acupuncture (STRICTA) standards to evaluate the quality of reporting for acupuncture interventions. By contrasting the findings of the meta-analysis with our own study, we may discern many parallels and contrasts that are pivotal for a thorough examination of the current data on acupuncture for post-stroke constipation. Firstly, the two studies may have discrepancies in their inclusion criteria and search methodologies. The study incorporated nine RCTs sourced from diverse databases, however the meta-analysis might have utilized distinct databases and a dissimilar time frame. These disparities may lead to inequalities in the quantity of research included and the characteristics of the patient population. Both studies evaluated several outcome measures including overall responder rate, constipation symptom scores, SP levels, latency to first bowel movement, serum VIP levels, and Bristol Stool Scale (BSS) scores. The findings of the two investigations shown congruency in some dimensions. Both studies demonstrated that acupuncture, whether used alone or in conjunction with conventional therapy, was superior to conventional therapy alone in enhancing the overall responder rate, decreasing constipation symptom scores, elevating SP levels, accelerating the time to the first bowel movement, and reducing VIP levels. Nevertheless, there were disparities in the outcomes pertaining to BSS scores. When acupuncture was used together with conventional therapy, it was found to be better than conventional therapy alone based on BSS scores. However, when acupuncture was used alone, there were no statistically significant differences compared to conventional therapy. The results of this meta-analysis have significant implications for the practical use of abdominal EA therapy in treating poststroke constipation. Incorporating abdominal EA into rehabilitation programs for stroke patients has the potential to provide a beneficial additional treatment method. Abdominal EA therapy has the potential to improve the QoL and overall recovery of poststroke patients by boosting gastrointestinal motility and alleviating constipation symptoms. Furthermore, the therapy is appealing for clinical practice due to its relatively affordable price and little negative effects.

EA has been recognized as a treatment method that provides benefits compared to other therapies and shows efficacy in treating constipation (25). Recognizing the constraints of our research is crucial. Initially, despite attempts to incorporate extensive datasets, it is not possible to completely eliminate the potential for publication bias. Furthermore, the studies that were considered may have intrinsic limitations, such as having small sample numbers or variances in study methods. Moreover, the variability among the trials may contribute to a certain level of ambiguity in the combined findings. Finally, this meta-analysis only considered papers written in English and Chinese languages, which may introduce a linguistic bias. It is important to consider these constraints while analyzing the results. In order to encourage the worldwide adoption of EA and assure the efficient implementation of medical scientific research findings, future RCTs should adhere to universal diagnostic criteria and efficacy evaluation criteria, guided by evidence-based medicine (48). In order to improve the quality of EA research, it is crucial to focus on patients’ TCM symptoms and apply tailored EA methods. Furthermore, in the process of conducting clinical research, it is essential to guarantee the appropriate randomization of cases in order to assure the comparability of the observation and control groups. However, researchers frequently neglect the extraction and analysis of TCM syndrome differentiation data while assessing the equilibrium of fundamental data sets. Since EA is based on TCM syndrome differentiation, the lack of data on syndrome differentiation makes it difficult to determine the comparability across groups. In clinical controlled research, the inclusion of a blank control group can aid in the unbiased evaluation of the effectiveness of acupuncture.

## 5 Conclusion

Overall, the findings of this meta-analysis indicate that EA as a therapeutic intervention enhances the overall effectiveness, rate of complete recovery, CCS, and QoL in stroke patients suffering from constipation, without causing any additional negative effects. Nevertheless, due to the absence of rigorous studies, the primary conclusions have been compromised in terms of their quality. In order to address the limitations of current research and provide strong evidence for the clinical use and recommendations of various types of EA, it is necessary to conduct further well-designed and high-quality trials with large sample sizes.

## Data Availability

The original contributions presented in the study are included in the article/supplementary material, further inquiries can be directed to the corresponding author.

## Footnotes

^1^ https://www.crd.york.ac.uk/PROSPERO
